# Mediation Analyses of the Mechanisms by Which Socioeconomic Status, Comorbidity, Stroke Severity, and Acute Care Influence Stroke Outcome

**DOI:** 10.1212/WNL.0000000000207939

**Published:** 2023-12-05

**Authors:** Anita Lindmark, Marie Eriksson, David Darehed

**Affiliations:** From the Department of Statistics (A.L., M.E.), Umeå School of Business, Economics and Statistics, and Sunderby Research Unit (D.D.), Department of Public Health and Clinical Medicine, Umeå University, Sweden.

## Abstract

**Background and Objectives:**

Low socioeconomic status (SES) is associated with increased risk of death and disability after stroke, but interventional targets to minimize disparities remain unclear. We aim to assess the extent to which SES-based disparities in the association between low SES and death and dependency at 3 months after stroke could be eliminated by offsetting differences in comorbidity, stroke severity, and acute care.

**Methods:**

This nationwide register-based cohort study included all 72 hospitals caring for patients with acute stroke in Sweden. All patients registered with an acute ischemic stroke in the Swedish Stroke Register in 2015–2016 who were independent in activities of daily living (ADL) during stroke were included. Data on survival and SES the year before stroke were retrieved by cross-linkage with other national registers. SES was defined by education and income and categorized into low, mid, and high. Causal mediation analysis was used to study the absolute risk of death and ADL dependency at 3 months depending on SES and to what extent hypothetical interventions on comorbidities, stroke severity, and acute care would equalize outcomes.

**Results:**

Of the 25,846 patients in the study, 6,798 (26.3%) were dead or ADL dependent 3 months after stroke. Adjusted for sex and age, low SES was associated with an increased absolute risk of 5.4% (95% CI 3.9%–6.9%; *p* < 0.001) compared with mid SES and 10.1% (95% CI 8.1%–12.2%; *p* < 0.001) compared with high SES. Intervening to shift the distribution of all mediators among patients with low SES to those of the more privileged groups would result in absolute reductions of these effects by 2.2% (95% CI 1.2%–3.2%; *p* < 0.001) and 4.0% (95% CI 2.6%–5.5%; *p* < 0.001), respectively, with the largest reduction accomplished by equalizing stroke severity.

**Discussion:**

Low SES patients have substantially increased risks of death and ADL dependency 3 months after stroke compared with more privileged patient groups. This study suggests that if we could intervene to equalize SES-related differences in the distributions of comorbidity, acute care, and stroke severity, up to 40 of every 1,000 patients with low SES could be prevented from dying or becoming ADL dependent.

## Introduction

Globally, stroke is the third-leading cause of death and disability, and lower-income and lower-middle–income countries carry the absolute majority of the stroke burden.^1^ Low socioeconomic status (SES), regardless of whether it is measured between or within countries, has repeatedly been linked to an increased risk of stroke, more severe strokes, and poor outcomes, including higher mortality and increased disability.^2-4^

Although it has also been demonstrated that patients with low SES have higher prevalence of cardiovascular risk factors and are underprivileged regarding quality of stroke care, access to stroke care, and secondary prevention after stroke,^5-10^ the mechanisms by which SES affects adverse stroke outcomes remain largely unknown. Previous studies using mediation analysis to explain the SES-adverse outcome relationship have found that stroke severity seems to be an important mediator—both in terms of short-term mortality^11^ and long-term disability,^12^ while quality of acute care was found to explain very little of the effect of SES on short-term mortality and readmission.^13^

In this nationwide register-based cohort study on stroke patients in Sweden, we use novel mediation analysis methods that allow for the evaluation of multiple mediators at once^14,15^ to investigate the connections between SES and adverse outcome (death and dependency) 3 months after stroke. We explore the extent to which SES disparities would remain if we could perform interventions to eliminate differences in comorbidity, stroke severity, and/or acute treatment.

## Methods

### Design and Setting

**Figure 1:**
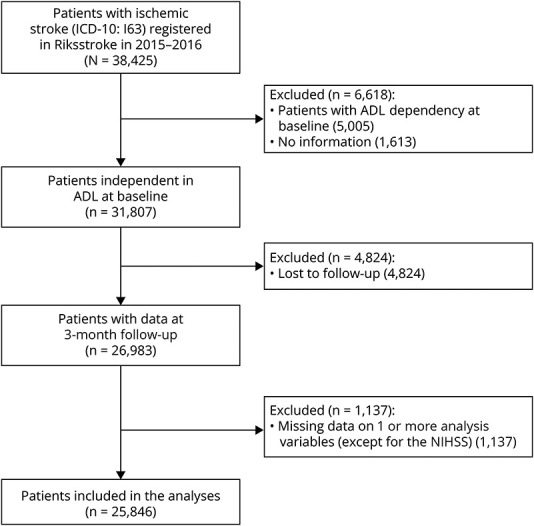
Flowchart Showing the Data Selection ADL = activities of daily living; NIHSS = NIH Stroke Scale.

This study included all patients registered as having had an acute ischemic stroke (*ICD-10*: code I63) in the Swedish Stroke Register (Riksstroke) from 2015 to 2016 who were independent in activities of daily living (ADL) before stroke onset (see study flowchart in Figure 1). The main aim of Riksstroke is to monitor stroke care quality in Sweden and to support improvements in said care, with a secondary aim of providing a database for stroke research.^16^ All 72 Swedish hospitals that provide acute stroke care report to the register, with a nationwide coverage in excess of 90% of all stroke patients treated in hospital.^17^ At the acute stage, data are prospectively registered by hospital staff. Patient-reported outcomes are collected at follow-ups 3 and 12 months after stroke.^16^

To obtain information on survival and SES, Riksstroke data were linked to the Swedish Cause of Death Register, managed by the Swedish National Board of Health and Welfare, and the Longitudinal Integrated Database for Health Insurance and Labor Market Studies, managed by Statistics Sweden. The registers were linked at the patient level using Swedish national identification numbers. The register holders performed the data linkage, and linked data were pseudonymized before being provided to us.

### Standard Protocol Approvals, Registrations, and Patient Consents

The study is covered by ethical approval from the regional ethics review board in Umeå, Sweden (reference number 2017/184-31). Patients were informed about registration in Riksstroke and about the register's aims. They were offered the right to decline participation (opt-out consent). According to the Swedish Patient Data Act, data from national quality registers may, after ethical approval, be processed for research purposes without a written consent. This study is reported in accordance with Strengthening the Reporting of Observational Studies in Epidemiology guidelines.^18^

### Variables

**Figure 2:**
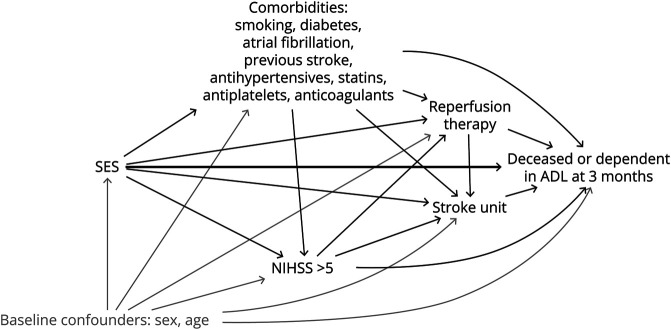
Directed Acyclic Graph of the Hypothesized Relationships Between the Study Variables ADL = activities of daily living; NIHSS = NIH Stroke Scale; SES = socioeconomic status.

A directed acyclic graph was used to discuss and describe the hypothesized relationships among the study variables (Figure 2).

#### Exposure: SES

SES was defined using a composite measure of education and income. Highest attained education level could assume 1 of 3 levels: primary school, secondary school, or university. Income was based on the individual's portion of the family's disposable income the year before the stroke and categorized into tertiles based on the unselected population (all stroke types, with and without follow-up data at 3 months, not limited to ADL independent at baseline) to better capture each individual's position in their peer group. The lowest tertile cutoffs were 155,033 SEK (Swedish krona) for patients registered in 2015 and 157,300 SEK for patients registered in 2016. The highest tertile cutoffs were 220,300 SEK for patients registered in 2015 and 229,700 SEK for patients registered in 2016. SES was categorized as follows: low (primary school education and income in the lowest tertile), high (university education and income in the highest tertile), and mid (everyone in between).

#### Mediators: Comorbidity, Stroke Severity, and Acute Care

Comorbidities include whether the patient had diabetes, atrial fibrillation, or previous stroke; which drugs the patient was prescribed at the time of stroke (antihypertensives, statins, antiplatelets, and anticoagulants); and smoking habits (smoker vs nonsmoker or unknown). Stroke severity was based on the NIH Stroke Scale (NIHSS) and dichotomized as mild stroke (NIHSS ≤5) and moderate-to-severe stroke (NIHSS >5). Acute care includes 2 separate variables: reperfusion therapies (thrombolysis and thrombectomy) and stroke unit care (defined as care in a stroke unit, neurosurgical ward, or intensive care unit at some point during the acute care episode).

#### Outcome: Death or ADL Dependency at 3 Months

A patient was classified as dead or ADL dependent at 3 months if they were either registered as ADL dependent at the 3-month follow-up or had died within 90 days of their stroke. ADL dependency was defined as patients who were unable to manage dressing, using the bathroom, or moving around indoors unassisted.

#### Baseline Confounders: Age and Sex

The baseline confounders included in the study were sex (male or female) and age at the time of stroke (years).

### Statistical Methods

All statistical analyses were performed using R^19^ version 4.1.2, and analysis code is available on GitHub.^20^ To obtain a general idea of the relationships in the directed acyclic graph (Figure 2), preliminary analyses were performed by fitting logistic regression models for the mediators, given the exposure and baseline confounders (sex and age), and the outcome, given the exposure, baseline confounders (sex and age), and mediators. Models are presented using odds ratios (ORs) and 95% CIs.

The total association between SES and death or ADL dependency at 3 months was estimated as the absolute difference between the risk of death or ADL dependency among patients with low SES and patients with mid and high SES, respectively, adjusted for the baseline confounders of sex and age. The absolute risk difference is presented with 95% CIs.

#### Mediation Analysis

To separate the effects of SES on death or ADL dependency at 3 months into direct vs indirect effects through the proposed mediators, we used causal mediation analysis.^21^ Where traditional approaches^22,23^ are limited to specific statistical models (often linear regression models), causal mediation analysis defines effects more generally and allow effects to be estimated in a wide variety of settings.^24^ Different causal mediation approaches can be used to address the complexity of multiple mediators.^15,25-27^ We used an approach estimating so-called “interventional disparity effects,”^14^ which has the advantage of not requiring strong assumptions regarding the causal ordering of the mediators.

Interventional disparity indirect effects correspond to the extent by which the total association would be eliminated if we could intervene to change the distributions of the mediators of patients with low SES to those of patients with mid SES and high SES, respectively.^14^ These effects were estimated for (1) intervening to shift the distributions of all mediators at once and (2) intervening to shift the distributions of the 4 mediators (comorbidities, severe stroke, reperfusion therapy, and stroke unit care) separately and for intervening to make the interdependence between the mediators in the low SES group the same as that of the mid or high SES groups. The interventional disparity direct effect corresponds to how much of the association between SES and death or ADL dependency at 3 months would remain if the distributions of the mediators were made to be the same in patients with low SES as in patients with mid or high SES.^14^ These effects are defined in more detail in the eMethods (links.lww.com/WNL/D211).

We used a simulation-based procedure frequently suggested for estimating effects in causal mediation analysis with multiple mediators^14,15,25,27,28^ to estimate the interventional disparity indirect and direct effects (for details see the eMethods, links.lww.com/WNL/D211). In brief, logistic regression models were built for the outcome, given the exposure, mediators, and confounders (sex and age), and for the mediators, given the subsets of preceding mediators, exposure, and confounders (sex and age).^14,15,27^ To reduce the risk of bias from model misspecification, we made the models flexible by including age-squared and all 2-way interactions between exposure and mediators, except between treatment with anticoagulants and reperfusion therapy because there were too few cases.^15^ Values were then simulated based on these models; this was repeated 200 times, and the effects were calculated by contrasting average predicted risks of outcome across all simulations. Bootstrap was used to estimate standard errors. Results are reported in accordance with recommendations in the A Guideline for Reporting Mediation Analyses statement.^29^

#### Missing Values

Proportions of missing values were generally small (<1.1%, Table 1), apart from the NIHSS, with almost 42% missing data. We therefore performed a complete case analysis (where patients with missing values on 1 or more variables were excluded) for all variables, except for the NIHSS. We used multiple imputation to replace the missing values of the dichotomized NIHSS variable with plausible imputed values. The underlying assumption for the multiple imputation is that NIHSS data are missing at random; that is, that given observed data, the risk of NIHSS data being missing does not depend on unobserved data.^30^ Numbers of missing NIHSS values for each category of the study variables are summarized in eTable 1 (links.lww.com/WNL/D207). Patients not receiving reperfusion therapy and not treated in stroke unit were much more likely to be missing the NIHSS.

**Table 1:**
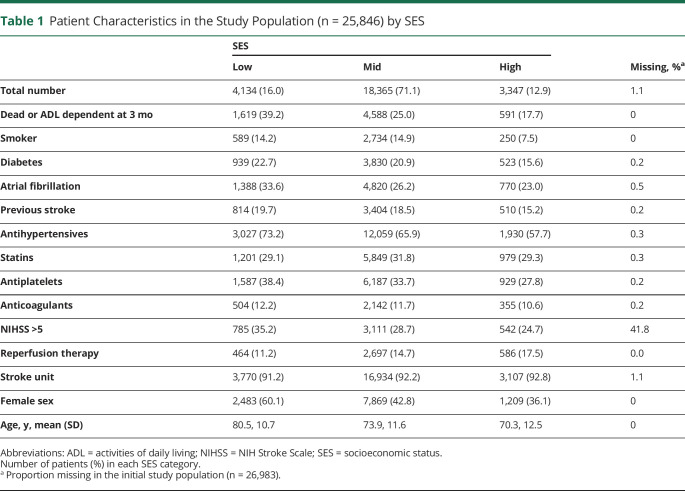
Patient Characteristics in the Study Population (n = 25,846) by SES

	SES			Missing, %^a^
	Low	Mid	High	
Total number	4,134 (16.0)	18,365 (71.1)	3,347 (12.9)	1.1
Dead or ADL dependent at 3 mo	1,619 (39.2)	4,588 (25.0)	591 (17.7)	0
Smoker	589 (14.2)	2,734 (14.9)	250 (7.5)	0
Diabetes	939 (22.7)	3,830 (20.9)	523 (15.6)	0.2
Atrial fibrillation	1,388 (33.6)	4,820 (26.2)	770 (23.0)	0.5
Previous stroke	814 (19.7)	3,404 (18.5)	510 (15.2)	0.2
Antihypertensives	3,027 (73.2)	12,059 (65.9)	1,930 (57.7)	0.3
Statins	1,201 (29.1)	5,849 (31.8)	979 (29.3)	0.3
Antiplatelets	1,587 (38.4)	6,187 (33.7)	929 (27.8)	0.2
Anticoagulants	504 (12.2)	2,142 (11.7)	355 (10.6)	0.2
NIHSS >5	785 (35.2)	3,111 (28.7)	542 (24.7)	41.8
Reperfusion therapy	464 (11.2)	2,697 (14.7)	586 (17.5)	0.0
Stroke unit	3,770 (91.2)	16,934 (92.2)	3,107 (92.8)	1.1
Female sex	2,483 (60.1)	7,869 (42.8)	1,209 (36.1)	0
Age, y, mean (SD)	80.5, 10.7	73.9, 11.6	70.3, 12.5	0

Abbreviations: ADL = activities of daily living; NIHSS = NIH Stroke Scale; SES = socioeconomic status.

Number of patients (%) in each SES category.

a Proportion missing in the initial study population (n = 26,983).

The imputation model used to impute the dichotomized NIHSS variable was a logistic regression model including all analysis variables and 3 auxiliary variables: level of consciousness based on the Reaction Level Scale (RLS = 1 vs RLS >1); whether the patient arrived to the hospital in an ambulance (yes/no/no information); and time from stroke onset to hospital arrival (<3, 3–<4.5, 4.5–<6, 6–24, >24 hours, no information). The imputation model included age-squared and all 2-way interactions between exposure, mediators, and outcome; except for the anticoagulant-reperfusion interaction.

Following recommendations that the number of imputed datasets should be at least equal to the percentage of missing data,^30^ we produced 45 imputed datasets that were then analyzed, and the results were pooled using Rubin's rules to obtain overall estimates. The R^19^ mice package^31^ was used for these imputations. The code used for the imputations can be found on GitHub.^20^ Diagnostics of the imputations can be found in the eMethods (links.lww.com/WNL/D211).

### Data Availability

Because of the sensitive nature of the data, supporting data are not publicly available. Requests for access to the dataset may be sent to Riksstroke at riksstroke@regionvasterbotten.se and require permissions from Statistics Sweden and the National Board of Health and Welfare (Registerservice@socialstyrelsen.se).

## Results

We identified 31,807 eligible patients, of whom 26,983 (84.8%) were either followed up or dead 3 months after stroke (Figure 1). Of them, 1,137 (4.2%) were excluded because of missing values on 1 or more of the analysis variables (except for the NIHSS). This left a final study population of 25,846 patients, with an average age of 74.4 years (SD = 11.9), 46.7% of whom were female. A total of 6,798 (26.3%) patients were dead or ADL dependent 3 months after stroke, with a higher risk of adverse outcome among patients with low SES, compared with mid or high SES (Table 1, eTable 2, links.lww.com/WNL/D208).

The proportion of female patients decreased with increasing SES (Table 1), while the average age was highest in the low SES group. Patients in lower SES groups had higher proportions of diabetes and atrial fibrillation and were more often prescribed antihypertensive and antiplatelet drugs than those in the high SES group, while differences in statin and anticoagulant treatments were negligible between different SES levels (Table 1, eTable 2, links.lww.com/WNL/D208).

Lower SES was associated with a higher risk of moderate-to-severe strokes (Table 1), and the proportions remained similar after imputation: median 35.1% (min–max 34.0%–36.5%) for patients with low SES, 27.1% (26.7%–27.6%) for patients with mid SES, and 22.9% (22.1%–23.8%) for patients with high SES. Acute care measurements including reperfusion therapy and treatment at stroke unit increased with increasing SES, although the differences for the latter were small (Table 1, eTable 2, links.lww.com/WNL/D208).

### Logistic Regression Models Adjusted for Confounders and Mediators

After adjustment for the baseline confounders sex and age (age + age-squared), low SES was associated with a higher risk of death or ADL dependency at 3 months, compared with both mid and high SES (Table 2, column 1). The associations remained but were reduced after further adjustments were made for mediators (Table 2, column 2).

**Table 2:**
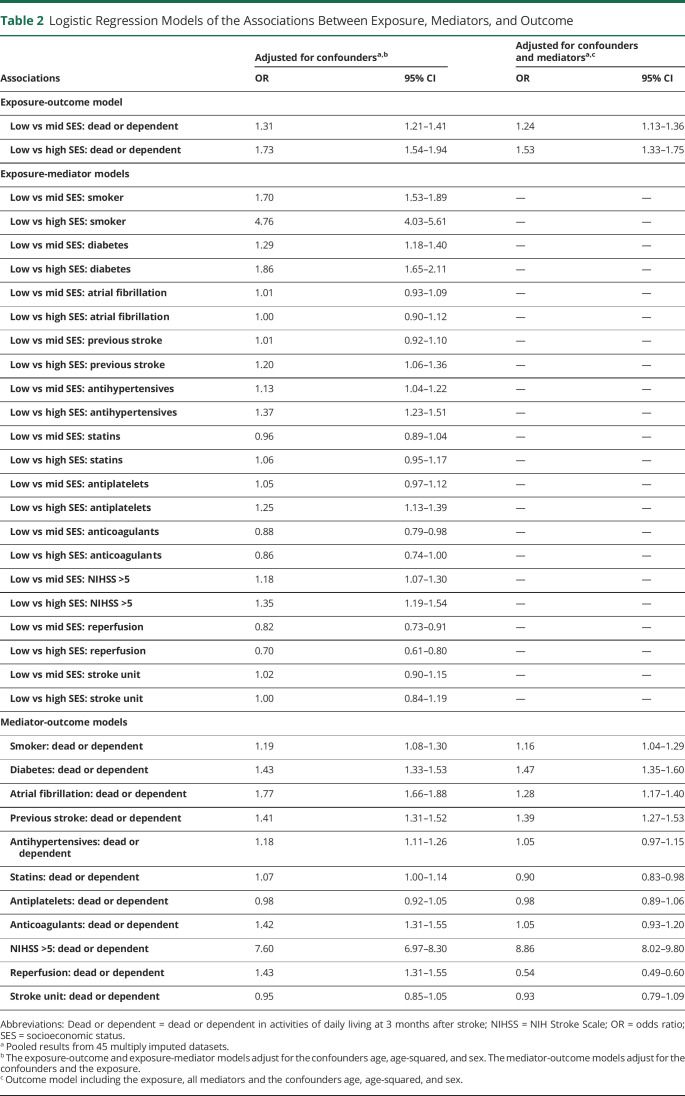
Logistic Regression Models of the Associations Between Exposure, Mediators, and Outcome

Associations	Adjusted for confounders^a,b^		Adjusted for confounders and mediators^a,c^	
	OR	95% CI	OR	95% CI
Exposure-outcome model				
Low vs mid SES: dead or dependent	1.31	1.21–1.41	1.24	1.13–1.36
Low vs high SES: dead or dependent	1.73	1.54–1.94	1.53	1.33–1.75
Exposure-mediator models				
Low vs mid SES: smoker	1.70	1.53–1.89	—	—
Low vs high SES: smoker	4.76	4.03–5.61	—	—
Low vs mid SES: diabetes	1.29	1.18–1.40	—	—
Low vs high SES: diabetes	1.86	1.65–2.11	—	—
Low vs mid SES: atrial fibrillation	1.01	0.93–1.09	—	—
Low vs high SES: atrial fibrillation	1.00	0.90–1.12	—	—
Low vs mid SES: previous stroke	1.01	0.92–1.10	—	—
Low vs high SES: previous stroke	1.20	1.06–1.36	—	—
Low vs mid SES: antihypertensives	1.13	1.04–1.22	—	—
Low vs high SES: antihypertensives	1.37	1.23–1.51	—	—
Low vs mid SES: statins	0.96	0.89–1.04	—	—
Low vs high SES: statins	1.06	0.95–1.17	—	—
Low vs mid SES: antiplatelets	1.05	0.97–1.12	—	—
Low vs high SES: antiplatelets	1.25	1.13–1.39	—	—
Low vs mid SES: anticoagulants	0.88	0.79–0.98	—	—
Low vs high SES: anticoagulants	0.86	0.74–1.00	—	—
Low vs mid SES: NIHSS >5	1.18	1.07–1.30	—	—
Low vs high SES: NIHSS >5	1.35	1.19–1.54	—	—
Low vs mid SES: reperfusion	0.82	0.73–0.91	—	—
Low vs high SES: reperfusion	0.70	0.61–0.80	—	—
Low vs mid SES: stroke unit	1.02	0.90–1.15	—	—
Low vs high SES: stroke unit	1.00	0.84–1.19	—	—
Mediator-outcome models				
Smoker: dead or dependent	1.19	1.08–1.30	1.16	1.04–1.29
Diabetes: dead or dependent	1.43	1.33–1.53	1.47	1.35–1.60
Atrial fibrillation: dead or dependent	1.77	1.66–1.88	1.28	1.17–1.40
Previous stroke: dead or dependent	1.41	1.31–1.52	1.39	1.27–1.53
Antihypertensives: dead or dependent	1.18	1.11–1.26	1.05	0.97–1.15
Statins: dead or dependent	1.07	1.00–1.14	0.90	0.83–0.98
Antiplatelets: dead or dependent	0.98	0.92–1.05	0.98	0.89–1.06
Anticoagulants: dead or dependent	1.42	1.31–1.55	1.05	0.93–1.20
NIHSS >5: dead or dependent	7.60	6.97–8.30	8.86	8.02–9.80
Reperfusion: dead or dependent	1.43	1.31–1.55	0.54	0.49–0.60
Stroke unit: dead or dependent	0.95	0.85–1.05	0.93	0.79–1.09

Abbreviations: Dead or dependent = dead or dependent in activities of daily living at 3 months after stroke; NIHSS = NIH Stroke Scale; OR = odds ratio; SES = socioeconomic status.

a Pooled results from 45 multiply imputed datasets.

b The exposure-outcome and exposure-mediator models adjust for the confounders age, age-squared, and sex. The mediator-outcome models adjust for the confounders and the exposure.

c Outcome model including the exposure, all mediators and the confounders age, age-squared, and sex.

Low SES was associated with an increased risk of most comorbidities compared with those with mid and high SES, except for atrial fibrillation and treatment with statins and anticoagulants (Table 2, column 1). For the other mediators, low SES was associated with an increased risk of more severe strokes and a decreased chance of reperfusion therapy, while effects pertaining to the stroke unit care variable were small.

Independent of sex, SES, age, and other mediators, smoking, a medical history of diabetes, atrial fibrillation, and previous stroke were associated with an increased risk of death or ADL dependency at 3 months, while the effects of prescribed medications and stroke unit care were smaller (Table 2, column 2). Stroke severity was associated with a strong independent increase in the risk of death or ADL dependency, while reperfusion therapy was associated with a decreased risk.

### Quantifying the Interventional Disparity Direct and Indirect Effects

After adjustment for sex and age (age + age-squared), low SES was associated with an increased absolute risk of death or ADL dependency at 3 months of 5.4% (95% CI 3.9%–6.9%) compared with mid SES and of 10.1% (8.1%–12.2%) compared with high SES, and just more than 60% of this increased risk would remain if all mediators were shifted to have the same distribution among patients with low SES as that of the more privileged patients (Table 3).

**Table 3:**
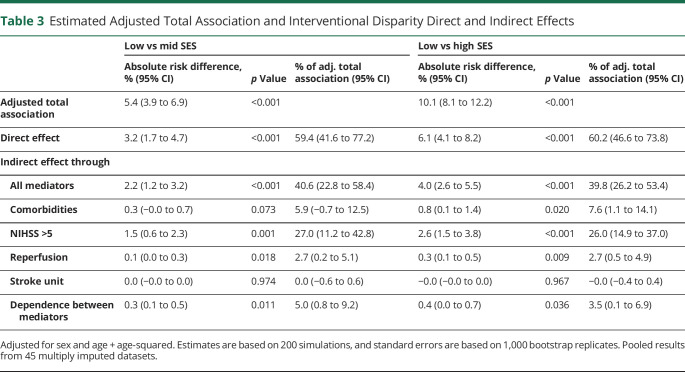
Estimated Adjusted Total Association and Interventional Disparity Direct and Indirect Effects

	Low vs mid SES			Low vs high SES		
	Absolute risk difference, % (95% CI)	*p* Value	% of adj. total association (95% CI)	Absolute risk difference, % (95% CI)	*p* Value	% of adj. total association (95% CI)
Adjusted total association	5.4 (3.9 to 6.9)	<0.001		10.1 (8.1 to 12.2)	<0.001	
Direct effect	3.2 (1.7 to 4.7)	<0.001	59.4 (41.6 to 77.2)	6.1 (4.1 to 8.2)	<0.001	60.2 (46.6 to 73.8)
Indirect effect through						
All mediators	2.2 (1.2 to 3.2)	<0.001	40.6 (22.8 to 58.4)	4.0 (2.6 to 5.5)	<0.001	39.8 (26.2 to 53.4)
Comorbidities	0.3 (−0.0 to 0.7)	0.073	5.9 (−0.7 to 12.5)	0.8 (0.1 to 1.4)	0.020	7.6 (1.1 to 14.1)
NIHSS >5	1.5 (0.6 to 2.3)	0.001	27.0 (11.2 to 42.8)	2.6 (1.5 to 3.8)	<0.001	26.0 (14.9 to 37.0)
Reperfusion	0.1 (0.0 to 0.3)	0.018	2.7 (0.2 to 5.1)	0.3 (0.1 to 0.5)	0.009	2.7 (0.5 to 4.9)
Stroke unit	0.0 (−0.0 to 0.0)	0.974	0.0 (−0.6 to 0.6)	−0.0 (−0.0 to 0.0)	0.967	−0.0 (−0.4 to 0.4)
Dependence between mediators	0.3 (0.1 to 0.5)	0.011	5.0 (0.8 to 9.2)	0.4 (0.0 to 0.7)	0.036	3.5 (0.1 to 6.9)

Adjusted for sex and age + age-squared. Estimates are based on 200 simulations, and standard errors are based on 1,000 bootstrap replicates. Pooled results from 45 multiply imputed datasets.

If we could intervene to shift the distribution of all mediators among patients with low SES to the distributions of those with higher SES, the absolute risk reduction in death or ADL dependency would be 2.2% (95% CI 1.2%–3.2%) compared with patients with mid SES and 4.0% (95% CI 2.6%–5.5%) compared with patients with high SES (Table 3). Much of this reduction among patients with low SES would be accomplished by intervening on stroke severity accounting for 1.5% (95% CI 0.6%–2.3%) and 2.6% (95% CI 1.5%–3.8%), respectively, of the increased absolute risk, compared with those with mid and high SES. Interventions focused on shifting the distributions of comorbidities, reperfusion therapy, and the dependence between mediators would yield smaller decreases in the absolute risk difference, while the indirect effects of stroke unit care were close to zero (Table 3).

## Discussion

This nationwide study showed that low SES was associated with a 5% increase in the absolute risk of death or ADL dependency 3 months after ischemic stroke compared with mid SES and a 10% increase compared with high SES. Approximately 40% of these excess risks were mediated through factors in the causal pathway, including comorbidities, stroke severity, and reperfusion therapy. This suggests that it could be possible to save 40 of every 1,000 patients in the low SES group from dying or becoming ADL dependent if we could equalize SES differences in comorbidity, stroke severity, and reperfusion therapy.

The increase in the risk of death and dependency for patients with low SES is in line with findings from previous studies on short-term mortality,^2,32-35^ disability,^36^ and the composite outcome of death or disability.^37^

Stroke severity was by far the most important mediator in this study. Previous studies have found that initial stroke severity explained approximately 40% of income inequalities in 3-month case fatality^11^ and more than 60% of income inequalities in long-term disability after ischemic stroke.^12^ We have previously studied the link between education level and stroke severity and found that nearly 30% of the effect was an indirect effect mediated through cardiovascular disease (CVD) risk factors (including smoking, diabetes, atrial fibrillation, previous stroke, and ADL dependency before the stroke).^38^ In this study, we found that part of the effect of SES on adverse outcome could be eliminated by only equalizing the distribution of comorbidities (risk factors and prescribed medications). Together with the likely importance of risk factors in the SES-stroke severity relationship, this means that an obvious target for clinical interventions aiming to reduce disparities in stroke outcomes would be to reduce disparities in comorbidities and risk factors of stroke among patients with low SES. Here, hypertension, atrial fibrillation, and diabetes are all important components related to lifestyle factors. The risks of hypertension and diabetes have been found to be modifiable by regular physical activity, a healthy diet, and weight loss, and hence, lifestyle changes including smoking cessation should be aggressively promoted, especially among those with low SES.^39,40^ Apart from physical inactivity and obesity, hypertension and diabetes can increase the risk of atrial fibrillation, and hence medication for hypertension and diabetes together with lifestyle changes are important in reducing the risk of atrial fibrillation.^41^

Previous studies have found that there is unequal access to acute stroke care across SES groups,^5,6,8,9^ and while differences in access to stroke unit care were small in our study, we found that there was unequal access to reperfusion treatment with patients with low SES less likely to receive reperfusion therapy. However, our results suggest that inequalities in adverse outcome at 3 months are not driven by inequalities in acute care. This is in line with a Danish mediation study on income inequalities in 30-day mortality and readmission, which found no mediating effect of quality of early care.^13^

The method used in the study relies on an assumption that there was no unobserved confounding of the mediator-outcome relationships (see the eMethods, links.lww.com/WNL/D211 for more details). Through linking individual registers, we were able to include several confounding factors and possible mediators. We were, however, limited to the variables collected by the registers. Functional outcome at 3 months is patient reported and based on a questionnaire and does not include the modified Rankin scale. However, ADL dependency based on questions in Riksstroke has shown good agreement with the modified Rankin scale,^42^ and with Barthel index,^43^ and we do not expect that this would have any major effects on the findings.

We had access to information on prescribed medications at the time of stroke (e.g., antihypertensives, statins) and CVD-related comorbidities (atrial fibrillation, diabetes, and previous stroke), but no or limited information on postacute care, patient preferences, lifestyle (e.g., alcohol consumption, physical activity), compliance with medications, other comorbidities (e.g., renal disease, heart failure, dementia, and cancer), other social determinants (e.g., occupation, neighborhood-level SES), or clinical measurements such as blood pressure or cholesterol levels. Furthermore, we did not consider stroke awareness and help-seeking behavior, factors that may lead to increased onset-to-door times and reduced benefit of reperfusion therapy. A previous review suggested that help-seeking behavior is more dependent on perceived severity of symptoms than on actual knowledge of symptoms and that delays were not related to sociodemographic factors.^44^ We were able to adjust for the major baseline confounders sex and age but cannot rule out residual confounding. Additionally adjusting for the hospital where the patient was treated as a sensitivity analysis for possible confounding by region did not have a major impact on the estimated logistic regression model parameters (eTable 3, links.lww.com/WNL/D209). An aim of future studies should be to broaden the included variables and mediators to further elucidate the complex relationship between SES and outcomes after stroke.

Stroke severity was measured using the NIHSS, dichotomized into mild stroke (0–5) and moderate-to-severe (>5) stroke. This dichotomization has been used in other studies, both as a predictor^45^ and as an outcome measure.^46^ While our results indicate that a substantial reduction in the death or ADL dependency disparity could be achieved by shifting the distribution of mild stroke (NIHSS 0–5) vs moderate-to-severe stroke (NIHSS >5) in patients with low SES to that of more privileged patient groups, it is possible that shifts based on a different cutoff or on a more fine-grained scale of the NIHSS could lead to reductions of a different magnitude. A more objective measure would be achieved by using imaging to gain information on infarct volume and location. Such information is not currently available in Riksstroke. NIHSS was missing for nearly half of the patients. However, we had extensive information on patient characteristics, level of consciousness at hospital admission, treatment, and outcome, which in combination with the choice to impute NIHSS to 2 categories rather than the full scale of measurement makes us expect no major deviations from the missing-at-random assumption. In addition, we used a flexible imputation model to reduce the risk of model misspecification. Under these conditions, a previous simulation study has shown that multiple imputation offers unbiased results, even with large proportions of missing data (up to 90% missing).^47^ Less than 5% of patients were missing data on variables other than the NIHSS. These patients were excluded.

The study is based on a nationwide quality register with high coverage. The analysis was restricted to patients who died or responded to the 3-month follow-up questionnaire. Nonresponders were more likely to be younger, have low SES, be smokers, have diabetes, or to have had a previous stroke, but were less likely to be prescribed antihypertensives or to receive reperfusion therapy (eTable 4, links.lww.com/WNL/D210). Selection may have biased the estimated absolute risk of death and dependency but is unlikely to have had a major impact on the main findings.

Although SES lacks a standard classification, it generally incorporates assessments of income, education, and/or occupation. These determinants are correlated but not interchangeable, and each measures different aspects of SES.^48^ For example, education is often established early in life and is considered a strong determinant of future income and occupation, while economic measures have been found to be more sensitive in detecting associations between SES and health, particularly in the nonelderly individuals.^49^ Composite measures have the potential to overcome some of the limitations of a single determinant. In this study, we used a composite measure of SES based on attained education and income. The combination of education and income into a composite measure has been shown to produce more comprehensive estimates of social inequalities in health.^50^

A limitation of our study is that no data on occupation were available, and hence, we could not capture, for example, aspects related to work-based psychosocial processes and environmental exposures.^48^ However, most of the patients were elderly individuals and likely to be retired, making occupation less important as a determinant of SES in our cohort. Finally, both education and income were obtained by register data and were therefore not subject to recall bias.

We used an approach to mediation analysis, which focuses on the reduction in observed SES disparities that could be accomplished by intervening to equalize the distributions of intermediate variables.^14^ One strength of this is that we shift the focus from infeasible interventions on SES itself to intervention targets that are more informative from a policy standpoint. In addition, the methods we used allow us to investigate effects of multiple mediators without making strong assumptions about the directions of associations between the mediators.^14,15^ The method used to estimate the effects relies on the specification of parametric regression models for the outcome and for the mediators. These models are subject to model misspecification bias. We tried to mitigate this issue by making the models as flexible as allowed by the data through the inclusion of interactions and age-squared.

Finally, the study setting was Sweden, a high-income country with publicly financed education and health care systems, and the generalizability of the study findings may be restricted to similar settings.

In our nationwide cohort study using prospectively collected data, we found that low SES was associated with an increased absolute risk of death and ADL dependency 3 months after stroke by 5%–10%, compared with higher SES. If we could intervene to minimize SES differences in comorbidity, stroke severity, and acute care, up to 40 of every 1,000 patients with low SES could potentially be saved from dying or becoming ADL dependent.

## Appendix Authors

**Table:**

Name	Location	Contribution
Anita Lindmark, PhD	Department of Statistics, Umeå School of Business, Economics and Statistics, Umeå University, Sweden	Drafting/revision of the article for content, including medical writing for content; study concept and design; and analysis and interpretation of data
Marie Eriksson, PhD	Department of Statistics, Umeå School of Business, Economics and Statistics, Umeå University, Sweden	Revision of the article for content; major role in the acquisition of data; study concept and design; and interpretation of data
David Darehed, MD, PhD	Sunderby Research Unit, Department of Public Health and Clinical Medicine, Umeå University, Sweden	Drafting/revision of the article for content, including medical writing for content; and study concept and design and interpretation of data

## Glossary

ADL: activities of daily living
CVD: cardiovascular disease
*ICD-10*: *International Classification of Diseases, Tenth Revision*
NIHSS: NIH Stroke Scale
OR: odds ratio
Riksstroke: Swedish Stroke Register
RLS: Reaction Level Scale
SES: socioeconomic status
